# lncRNA HCG11 suppresses human osteosarcoma growth through upregulating p27 Kip1

**DOI:** 10.18632/aging.203517

**Published:** 2021-09-13

**Authors:** Jie Gu, Bo Dai, Xuchao Shi, Zhennian He, Yuanlin Xu, Xiangqian Meng, Junlan Zhu

**Affiliations:** 1 Department of Orthopaedics Surgery, Beilun People's Hospital, Ningbo, Zhejiang, China; 2 Department of Stomatology, Beilun People's Hospital, Ningbo, Zhejiang, China; 3 The Precision Medicine Laboratory, Beilun People's Hospital, Ningbo, Zhejiang, China

**Keywords:** osteosarcoma, long non-coding RNA, human leukocyte antigen complex group 11, p27 Kip1, miR-942-5p, IGF2BP2

## Abstract

Osteosarcoma (OS) is a common malignant bone cancer threatening children and young adults. Emerging evidence indicates that long non-coding RNAs (lncRNAs) play crucial roles in the progression of OS. Herein, we want to clarify the roles of lncRNA human leukocyte antigen complex group 11 (HCG11) in OS. Our data revealed that HCG11 expression is decreased in OS, which is a result of transcriptional repression of YY1. Low HCG11 level is closely associated with larger tumor size and shorter overall survival of OS patients. HCG11 negatively regulates cell proliferation, cell cycle, DNA replication *in vitro* and tumor growth *in vivo*. HCG11 can raise p27 Kip1 expression via binding to miR-942-5p and IGF2BP2, and p27 Kip1 acts as a key effector for HCG11 exerting biological functions. In conclusion, HCG11 is downregulated in OS, and restrains OS growth both *in vitro* and *in vivo* by raising p27 Kip1 expression via binding to miR-942-5p and IGF2BP2.

## INTRODUCTION

Osteosarcoma (OS) is a common malignant bone cancer that mainly occurs during childhood and adolescence [1, 2]. OS features a highly malignant phenotype such as uncontrolled growth and invasiveness [3]. Although advanced therapies such as surgery combined with multiple chemotherapies have been applied in clinical practice, the outcomes and survival rates of OS patients remain unsatisfactory [4]. Therefore, identifying diagnostic markers and efficient therapeutic targets for OS are urgently needed. Before that, a better understanding of the molecular mechanisms, especially the complex gene regulation networks, of OS carcinogenesis is vital important.

Long non-coding RNAs (lncRNAs) are a class of non-coding RNA longer than 200 nucleotides in length [5]. They used to be considered as nonfunctional, while recent studies have shown that lncRNAs play key roles in various aspects of cell biological behavior, including metabolism, proliferation, differentiation, apoptosis and migration [6, 7]. LncRNAs exert their biological functions via diversified molecular mechanisms such as epigenetic regulation, transcriptional regulation, mRNA post-transcriptional modulation and proteins or microRNAs interaction [8, 9]. In OS, there also have been many studies devoted to investigate the roles of lncRNAs, and identified them as potential markers for diagnostics and therapeutics [10, 11].

HCG11 has been verified to act as a tumor suppressor gene in many cancers, including oral squamous cell carcinoma, non-small cell lung cancer, cervical cancer, laryngeal carcinoma, glioma and prostate cancer [12–17]. However, in some cancers, such as hepatocellular carcinoma and gastric cancer, HCG11 has been validated to be an oncogene [18, 19]. Until now, the expression, biological functions and the underlying regulatory mechanism of HCG11 have never been investigated in OS. Hence, the present study set out to explore and elucidate the roles of HCG11 in OS.

## RESULTS

### HCG11 expression is decreased in OS and closely related to clinical factors

**Figure 1:**
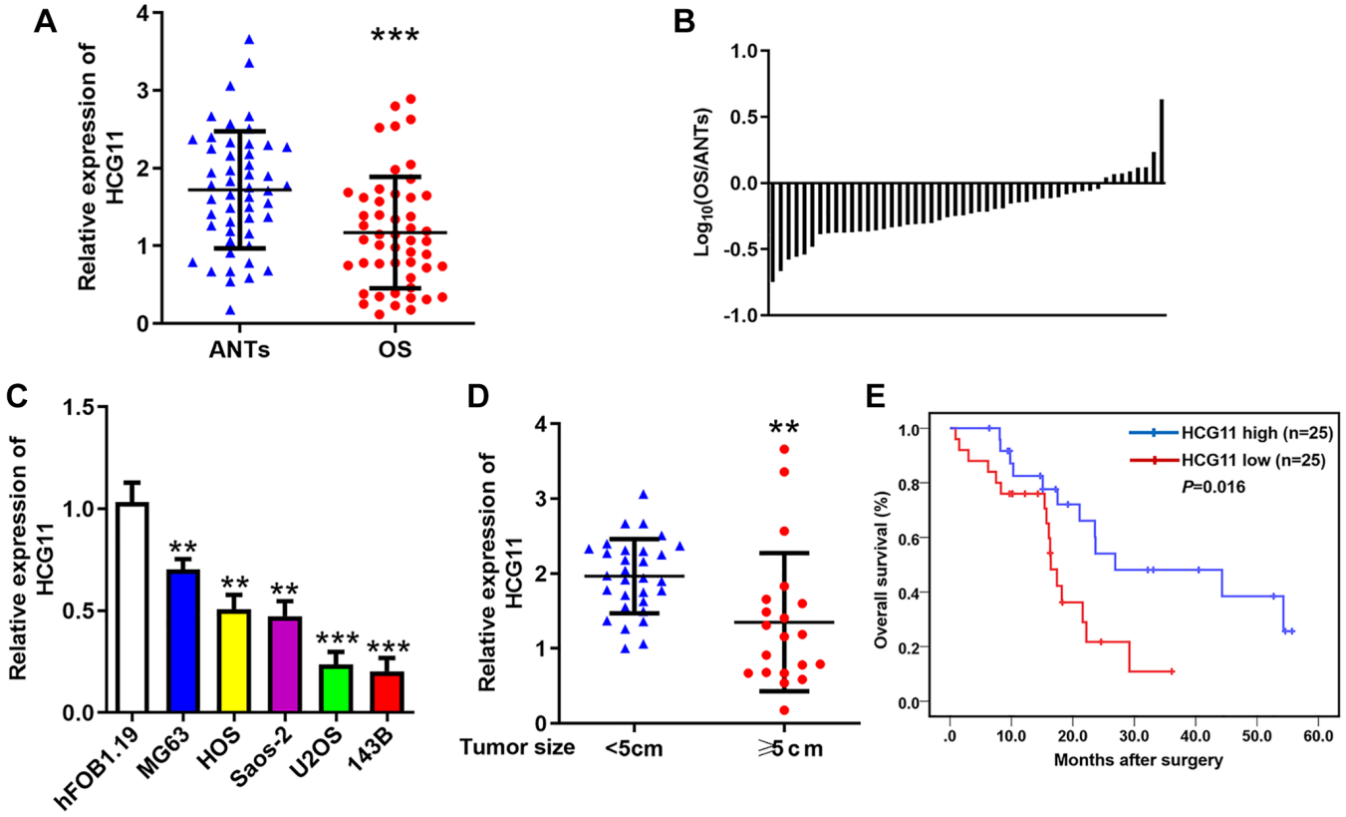
**HCG11 expression is decreased in OS and closely related to clinical factors.** (**A**) HCG11 expression in OS tissues and ANTs was analyzed by qRT-PCR. (**B**) The ratio of relative HCG11 expression in OS tissues versus ANTs shown on the logarithmic scale. (**C**) HCG11 expression in OS cell lines (MG63, HOS, Saos-2, U2OS and 143B) and human osteoblast cell line (hFOB1.19) was analyzed by qRT-PCR. (**D**) HCG11 level in tumors ≥ 5cm and < 5cm was compared. (**E**) Overall survival of OS patients was analyzed by the Kaplan-Meier method. ^**^P < 0.01, ^***^P < 0.001.

To identify the expression pattern of HCG11 in OS, qRT-PCR was performed to evaluate HCG11 expression in OS tissues and cell lines. We found that HCG11 expression is significantly lower in OS tissues than that in ANTs (Figure 1A and 1B), and also lower in OS cell lines than that in human osteoblast cell line (Figure 1C). After evaluating the association of HCG11 with clinicopathological parameters, we noticed that HCG11 expression is correlated with tumor size, while not significantly associated with age, gender, histological subtype, anatomic location, clinical stage, and distant metastasis (Table 1). We then showed that HCG11 level is markedly lower in patients with tumor size greater than or equal to 5cm (Figure 1D). Moreover, low HCG11 level also indicates shorter overall survival of OS patients (Figure 1E).

**Table 1:** Relationship between clinicopathological parameters and HCG11 expression.

**Variables**	**N**	**HCG low**	**HCG high**	***P***	**miR-942-5p low**	**miR-942-5p high**	***P***
**Gender**							
**Male**	28	14	14		13	15	
**Female**	22	11	11	0.612	12	10	0.388
**Age**							
**<25y**	30	15	15		14	16	
**≥25y**	20	10	10	0.613	11	9	0.387
**Tumor size**							
**<5cm**	29	8	21		19	10	
**≥5cm**	21	17	4	**<0.001**	6	11	**0.010**
**Lung metastasis**							
**No**	34	15	19		20	14	
**Yes**	16	10	6	0.182	5	11	0.064
**Enneking stage**							
**I + IIA**	31	14	17		18	13	
**IB + III**	19	11	8	0.280	7	12	0.122
**Differentiation grade**							
**Well/moderately**	30	14	16		15	15	
**Poorly/undifferentiated**	20	11	9	0.387	10	10	0.613
**Location**							
**Femur/tibia**	33	18	15		17	16	
**Elsewhere**	17	7	10	0.276	8	9	0.500

*P* < 0.05 was considered statistically significant (Chi-square test).

### Low HCG11 expression in OS is a result of transcriptional repression of YY1

**Figure 2:**
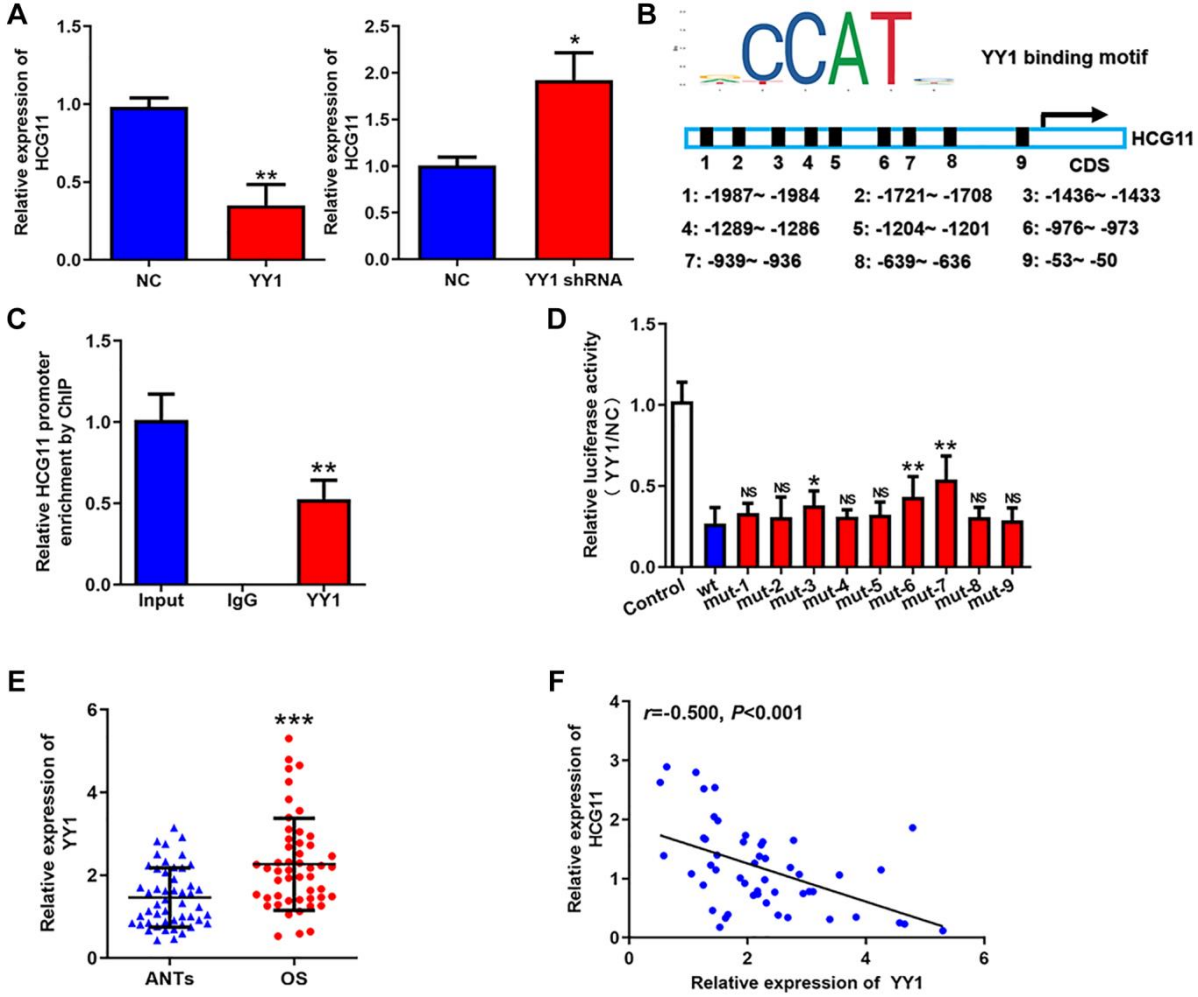
**Low HCG11 expression in OS is a result of transcriptional repression of YY1.** (**A**) HCG11 expression in OS cells detected by qRT-PCR after YY1 overexpression or knockdown. (**B**) Potential YY1 recognition sites in the promoter region of HCG11. (**C**) The combination of YY1 to the promoter region of HCG11 was validated by ChIP assay. (**D**) Several predicted binding sites were verified by luciferase assays. (**E**) YY1 mRNA expression in OS tissues and ANTs was analyzed by qRT-PCR. (**F**) Correlation between YY1 expression and HCG11 expression was analyzed by Spearman’s analysis. NS, no significance, ^*^P < 0.05, ^**^P < 0.01, ^***^P < 0.001.

Then, we attempted to clarify the mechanism of HCG11 downregulation. We found that overexpressing YY1 repressed HCG11 expression and silencing YY1 raised HCG11 expression (Figure 2A). Then, we further investigated the regulation of YY1 on HCG11. Through JASPAR database, we noticed that there are several YY1 recognition sites in the promoter region of HCG11 (Figure 2B). Because YY1 is a well-known transcriptional repressor and has already been verified to be highly expressed in OS [20–22], we wondered whether the downregulation of HCG11 is transcriptionally repressed by YY1. The ChIP assay suggested that YY1 binds to the promoter region of HCG11 (Figure 2C). The result of luciferase assay showed that YY1 restrained the luciferase activity of the reporter containing wild-type HCG11 promoter, while mutated some sites significantly attenuated the inhibitory effect (Figure 2D). These data demonstrate that YY1 suppresses HCG11 transcription by binding to its promoter region. In addition, we displayed that YY1 expression is upregulated in OS tissues and negatively correlated with HCG11 expression (Figure 2E and 2F), which further confirms the above conclusion.

### HCG11 is a negative regulator on OS growth *in vitro* and *in vivo*

**Figure 3:**
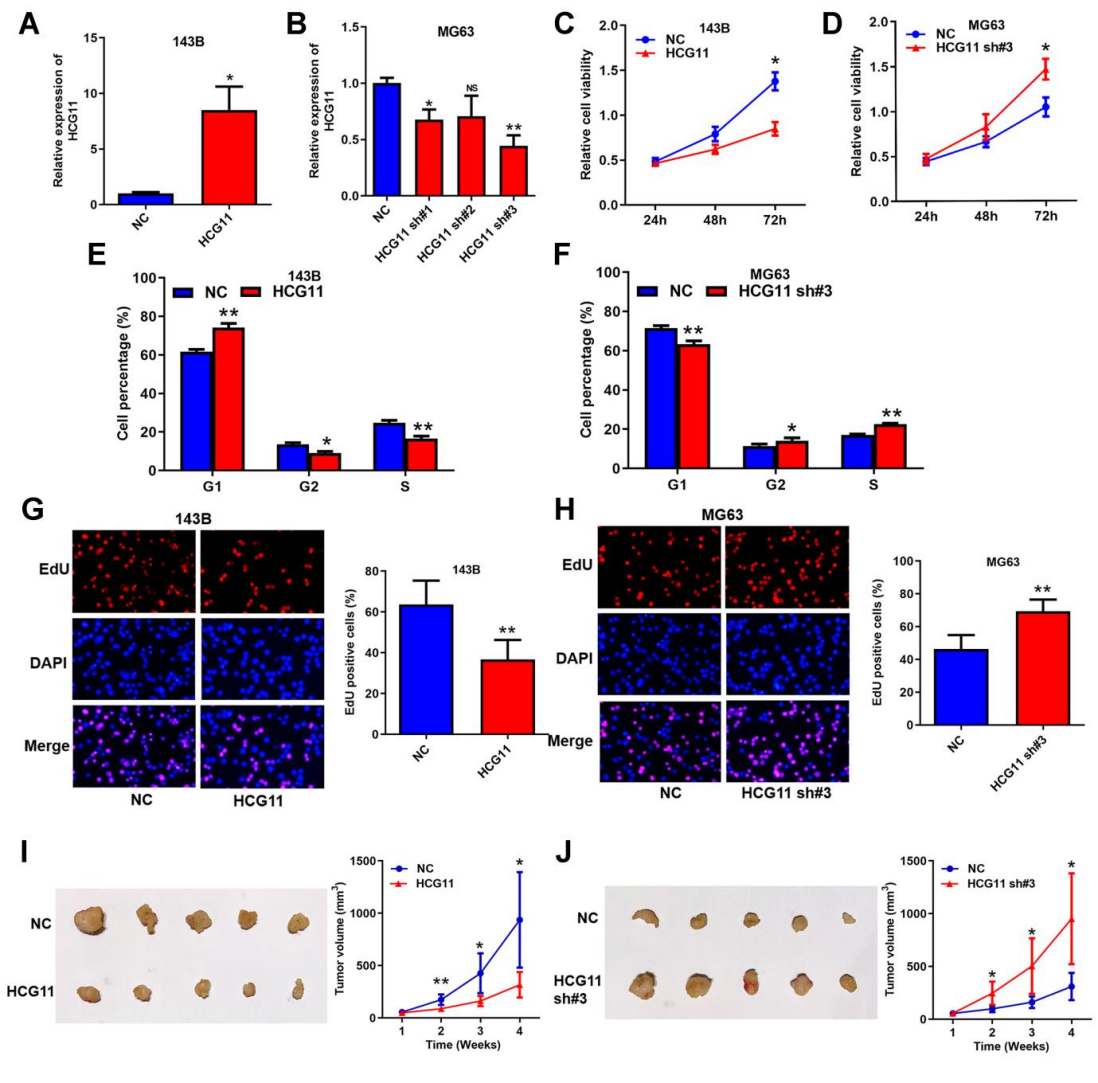
**HCG11 is a negative regulator on OS growth *in vitro* and *in vivo*.** (**A**, **B**) Overexpression and silence of HCG11 in OS cells validated by qRT-PCR. (**C**, **D**) Influence of HCG11 on the proliferation of OS cells was assessed by CCK8 assays. (**E**, **F**) Influence of HCG11 on the cell cycle of OS cells was analyzed by flow cytometry. (**G**, **H**) Influence of HCG11 on the DNA replication of OS cells was assessed by EdU assay. (**I**, **J**) Influence of HCG11 on OS tumor growth *in vivo* was estimate by Xenograft tumorigenesis. NS, no significance, ^*^P < 0.05, ^**^P < 0.01.

To investigate the biological functions of HCG11 in OS, it was successfully overexpressed in 143B cells and silenced in MG63 cells (shRNA#3 provided the most effective knockdown) (Figure 3A and 3B). Then, CCK8 assays showed that the proliferation capabilities of OS cells decreased upon transfection of HCG11 overexpression plasmids and increased after being transfected with shRNAs (Figure 3C, 3D, and Supplementary Figure 1A). Moreover, cell cycle analysis indicated that overexpressing HCG11 increased the cell proportion of G1 phase, and decreased the cell proportion of G2 and S phase (Figure 3E). Silencing HCG11 downregulated the cell proportion of G1 phase, and raised the cell proportion of G2 and S phase (Figure 3F and Supplementary Figure 1B). EdU assays showed that DNA replication activity was inhibited by HCG11 overexpression and promoted by HCG11 knockdown (Figure 3G, 3H, and Supplementary Figure 1C). Accordingly, the tumor growth *in vivo* was significantly restrained by HCG11 overexpression and accelerated by HCG11 knockdown (Figure 3I and 3J). Together, these results indicate that HCG11 is a negative regulator on OS growth *in vitro* and *in vivo*, which is also consistent with the fact that HCG11 level is negatively correlated with tumor size of OS patients (Table 1).

### HCG11 raises p27 Kip1 expression via binding to miR-942-5p and IGF2BP2

**Figure 4:**
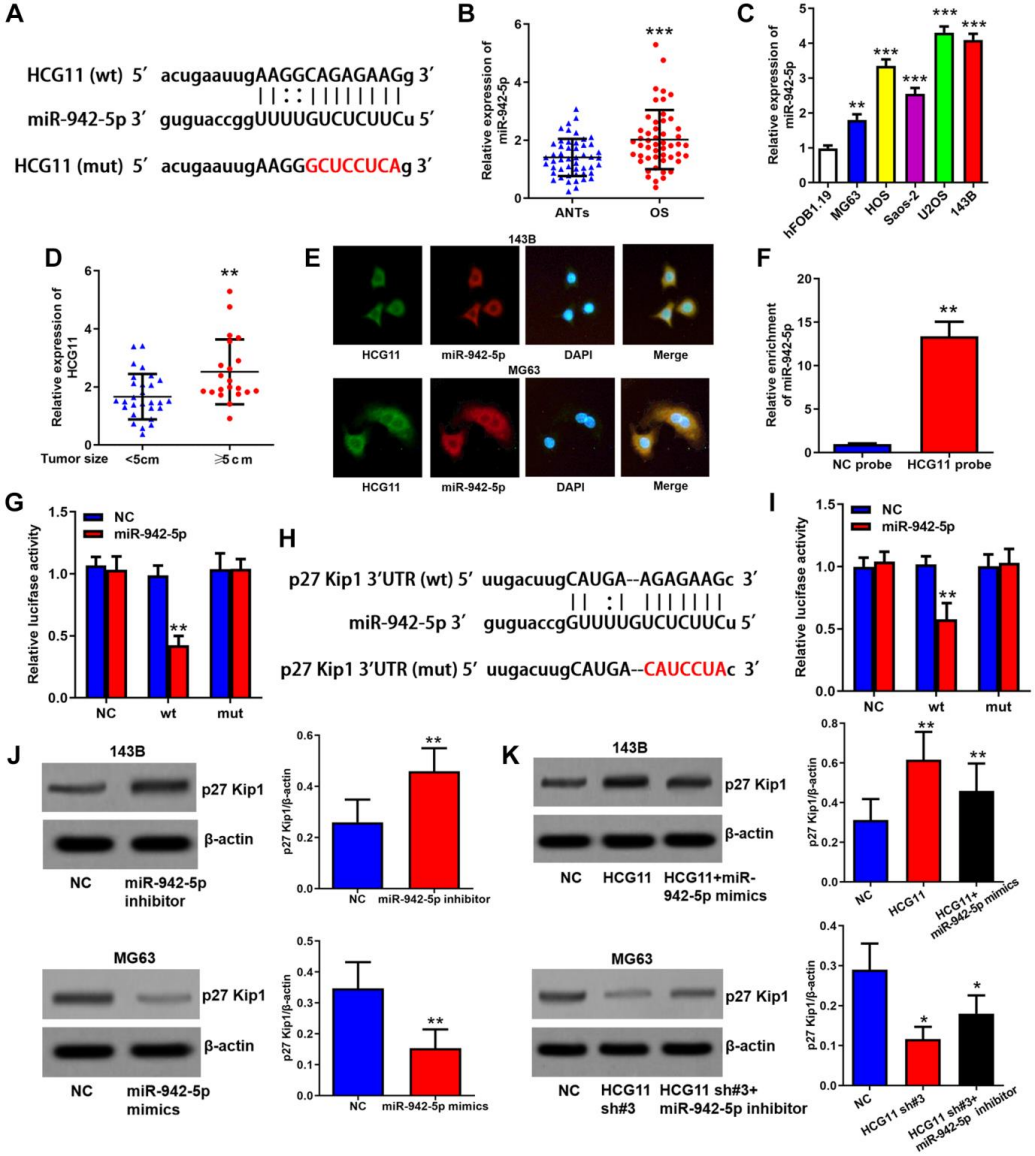
**HCG11 raises p27 Kip1 expression via binding to miR-942-5p.** (**A**) Predicted binding site of miR-942-5p in HCG11 sequence by StarBase database. (**B**) MiR-942-5p expression in OS tissues and ANTs was analyzed by qRT-PCR. (**C**) MiR-942-5p expression in OS cell lines and human osteoblast cell line was analyzed by qRT-PCR. (**D**) MiR-942-5p level in tumors ≥ 5cm and < 5cm was compared. (**E**) Subcellular co-localization of HCG11 and miR-942-5p was confirmed by RNA *in situ* hybridization assay. (**F**) Integration of HCG11 and miR-942-5p was proved by RNA pull-down assay. (**G**) Specific binding site of miR-942-5p in HCG11 were verified by luciferase assays. (**H**) Predicted binding site of miR-942-5p in p27 Kip1 3’UTR by StarBase database. (**I**) Specific binding site of miR-942-5p in p27 Kip1 3’UTR were verified by luciferase assays. (**J**) Regulation of miR-942-5p on p27 Kip1 protein level was estimated by western blot assay. (**K**) Regulation of HCG11 via inhibiting miR-942-5p on p27 Kip1 protein level was estimated by western blot assay. ^**^P < 0.01, ^***^P < 0.001.

Subsequently, we explored the molecular mechanisms of HCG11 in regulating OS growth. Through StarBase database (http://starbase.sysu.edu.cn/), we found miR-942-5p, which has already been reported to accelerate the growth of OS cells [23], may be a target of HCG11 (Figure 4A). Subsequently, we found that miR-942-5p expression is increased in OS tissues and cell lines, and positively correlated with tumor size of OS patients (Figure 4B–4D, Table 1). The images of RNA *in situ* hybridization assay indicated that both HCG11 and miR-942-5p are abundant in the cytoplasm of OS cells (Figure 4E). Then, data of RNA pull-down assay confirmed the integration of HCG11 and miR-942-5p (Figure 4F). We also performed a luciferase reporter assay to validate the predicted specific binding sites (Figure 4G). These data suggest that miR-942-5p is a direct target of HCG11. Furthermore, we found p27 Kip1 which is a G1 phase arrestor may be a potential target gene of miR-942-5p (Figure 4H). This combination was also validated by the luciferase reporter assay (Figure 4I). We showed that miR-942-5p is able to restrain p27 Kip1 protein level (Figure 4J) and HCG11 can raise p27 Kip1 protein expression via inhibiting miR-942-5p (Figure 4K).

**Figure 5:**
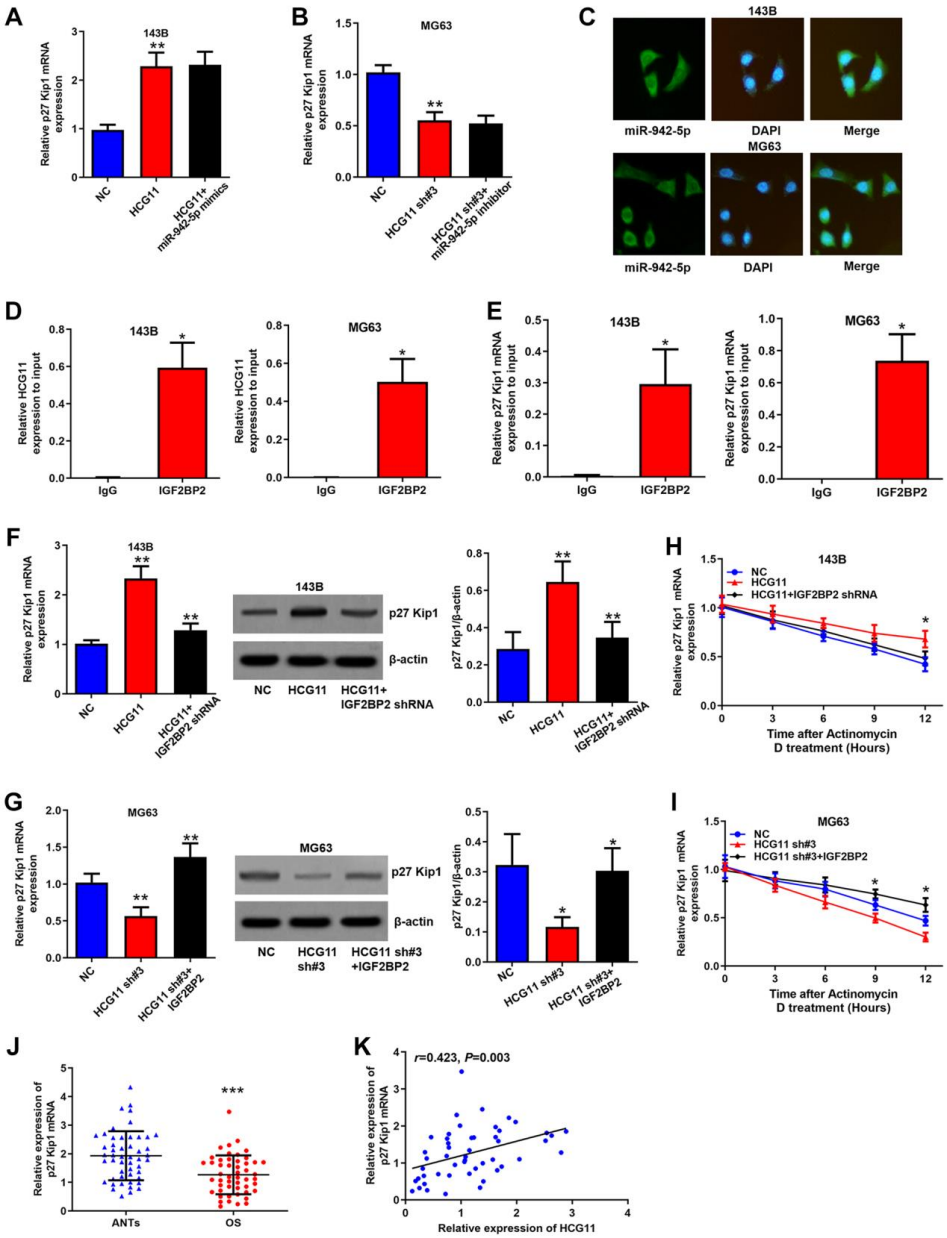
**HCG11 raises p27 Kip1 expression via binding to IGF2BP2.** (**A**, **B**) Regulation of HCG11 and miR-942-5p on p27 Kip1 mRNA level was detected by qRT-PCR. (**C**) Subcellular localization of IGF2BP2 was confirmed by immunofluorescence. (**D**, **E**) Integration of HCG11 and p27 Kip1 mRNA with IGF2BP2 was proved by RIP assay. (**F**, **G**) Regulation of HCG11 and IGF2BP2 on p27 Kip1 mRNA and protein level was estimated by qRT-PCR and western blot. (**H**, **I**) Influence of HCG11 and IGF2BP2 on p27 Kip1 mRNA degradation was estimated by RNA stability analysis. (**J**) p27 Kip1 mRNA expression in OS tissues and ANTs was analyzed by qRT-PCR. (**K**) Correlation between p27 Kip1 mRNA expression and HCG11 expression was analyzed by Spearman’s analysis. ^*^P < 0.05, ^**^P < 0.01, ^***^P < 0.001.

Interestingly, we found that HCG11 upregulated p27 Kip1 mRNA expression as well (Figure 5A and 5B). As we know, miRNAs bind to the 3′UTR region of mRNAs and inhibit the translation of target genes, not affecting the level of mRNAs. Here, we also demonstrated that the regulation of HCG11 on p27 Kip1 mRNA is unaffected by miR-942-5p (Figure 5A and 5B). So, how does HCG11 influence p27 Kip1 mRNA expression? Through StarBase database, we identified that both HCG11 and p27 Kip1 mRNA can bind to IGF2BP2. IGF2BP2 is a RNA-binding protein that is able to shield RNAs from endonucleases or microRNA-mediated degradation [24]. We speculated that HCG11 recruits IGF2BP2 to binding to p27 Kip1 mRNA and thereby inhibited its degradation. To verify this hypothesis, first, we showed that IGF2BP2 is abundant in the cytoplasm, displaying the same subcellular localization with HCG11 and p27 Kip1 mRNA (Figure 5C). Subsequently, RIP assay clearly demonstrated the enrichment of HCG11 and p27 Kip1 mRNA in the IGF2BP2 immunoprecipitates (Figure 5D and 5E). Moreover, the HCG11 overexpression induced p27 Kip1 mRNA and protein upregulation was attenuated by IGF2BP2 knockdown; HCG11 silence induced p27 Kip1 mRNA and protein downregulation was reversed by IGF2BP2 overexpression (Figure 5F and 5G). RNA stability analysis showed that overexpression or silence of HCG11 influenced the stability of p27 Kip1 mRNA, and these effects were abated by IGF2BP2 knockdown or upregulation, respectively (Figure 5H and 5I). In addition, we found that p27 Kip1 mRNA expression is significantly downregulated in OS tissues and positively correlated with HCG11 expression (Figure 5J and 5K). These data all together manifest that HCG11 also upregulates p27 Kip1 expression by recruiting IGF2BP2 to stabilize p27 Kip1 mRNA.

### p27 Kip1 is a key effector for HCG11 exerting biological functions

**Figure 6:**
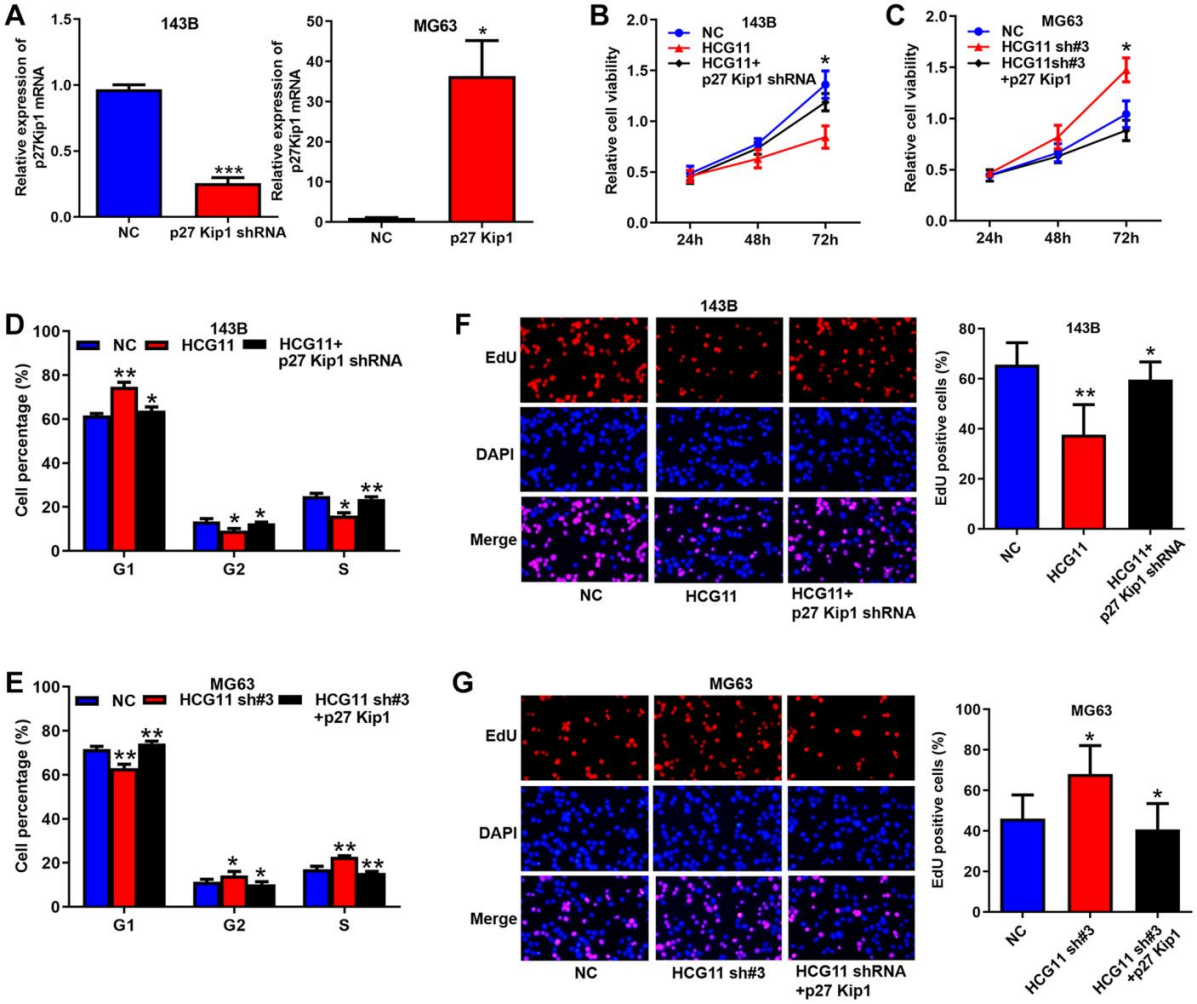
**p27 Kip1 is a key effector for HCG11 exerting biological functions.** (**A**) Overexpression and silence of p27 Kip1 in OS cells validated by qRT-PCR. (**B**, **C**) Influence of HCG11 and p27 Kip1 on the proliferation OS cells was assessed by CCK8 assays. (**D**, **E**) Influence of HCG11 and p27 Kip1 on the cell cycle of OS cells was analyzed by flow cytometry. (**F**, **G**) Influence of HCG11 and p27 Kip1 on the DNA replication of OS cells was assessed by EdU assay. ^*^P < 0.05, ^**^P < 0.01.

Finally, we investigated the roles of p27 Kip1 in HCG11 mediated OS growth (Figure 6A–6G). First, we successfully overexpressed and knocked-down p27 Kip1 in OS cells (Figure 6A). Then, CCK8 assay, cell cycle analysis and EdU assay discovered that the influence of HCG11 overexpression on cell proliferation, cell cycle and DNA replication was significantly abated by p27 Kip1 knockdown (Figure 6B, 6D and 6F). Likewise, inhibitory effects of HCG11 silencing were significantly restored by p27 Kip1 overexpression (Figure 6C, 6E and 6G). Altogether, these findings suggest that p27 Kip1 acts as a key effector for HCG11 exerting biological functions.

## DISCUSSION

Recently, an increasing number of studies have been devoted to clarifying the pivotal roles of lncRNAs in cancers, including OS. With regard to HCG11, it has been identified to be aberrantly expressed in some cancers and function as an oncogene or a tumor suppressor gene [12–19], not including OS yet. Consistent with most of the tumors reported, our data suggest that HCG11 is downregulated and exerts a tumor suppressive role in OS.

Here, we not only present the downregulation of HCG11 in OS, but also its clinical significance and clarified the mechanism of low expression. We show that low HCG11 expression is closely associated with larger tumor size and shorter overall survival of OS patients, which indicates that HCG11 is a potential biomarker for OS. In addition, we display that the low HCG11 expression is due to the transcriptional repression of YY1. YY1 is a well-known transcriptional repressor and has already been verified to be highly expressed in OS [20–22]. In this study, we demonstrate that YY1 suppresses HCG11 transcription by binding to its promoter region. As a result, the data that HCG11 is downregulated in OS will be more convincing.

In terms of biological function, we demonstrate that HCG11 suppresses OS growth both *in vitro* and *in vivo*. We show that HCG11 is able to arrest the cell cycle progression and restrain DNA replication, resulting in the slowdown of cell proliferation. These findings reveal that HCG11 acts as a tumor suppressor gene in OS, which is a good explanation for its close association with tumor size and overall survival.

When it comes to molecular mechanisms, we show that HCG11 exerts tumor suppressor roles by raising the p27 Kip1 expression. One possible approach is the common competing endogenous RNAs (ceRNA) manner in which lncRNAs abolish the suppressive effect of miRNAs on mRNAs by competitively binding with miRNAs [25]. Here, HCG11 increases p27 Kip1 expression by repressing the activity of miR-942-5p. MiR-942-5p has been proved to be upregulated and serve as an oncogene in many other cancers, including colorectal cancer, breast cancer, cervical cancer, non-small cell lung cancer, and so on [14, 26–28]. In OS, miR-942-5p have already been reported to accelerate the growth of OS cells [23]. In addition, we discover that miR-942-5p expression is increased in OS tissues and cell lines, and positively correlated with tumor size of OS patients, which adds the knowledge of miR-942-5p in OS. Another way for HCG11 raising p27 Kip1 expression is through recruiting IGF2BP2 to stabilize p27 Kip1 mRNA. This is also a possible mode for lncRNAs post-transcriptionally modulating mRNAs [29, 30]. IGF2BP2, which is a RNA-binding protein that can shield RNAs from endonucleases or microRNA-mediated degradation [24], acts as a key mediating molecule in this axis.

**Figure 7:**
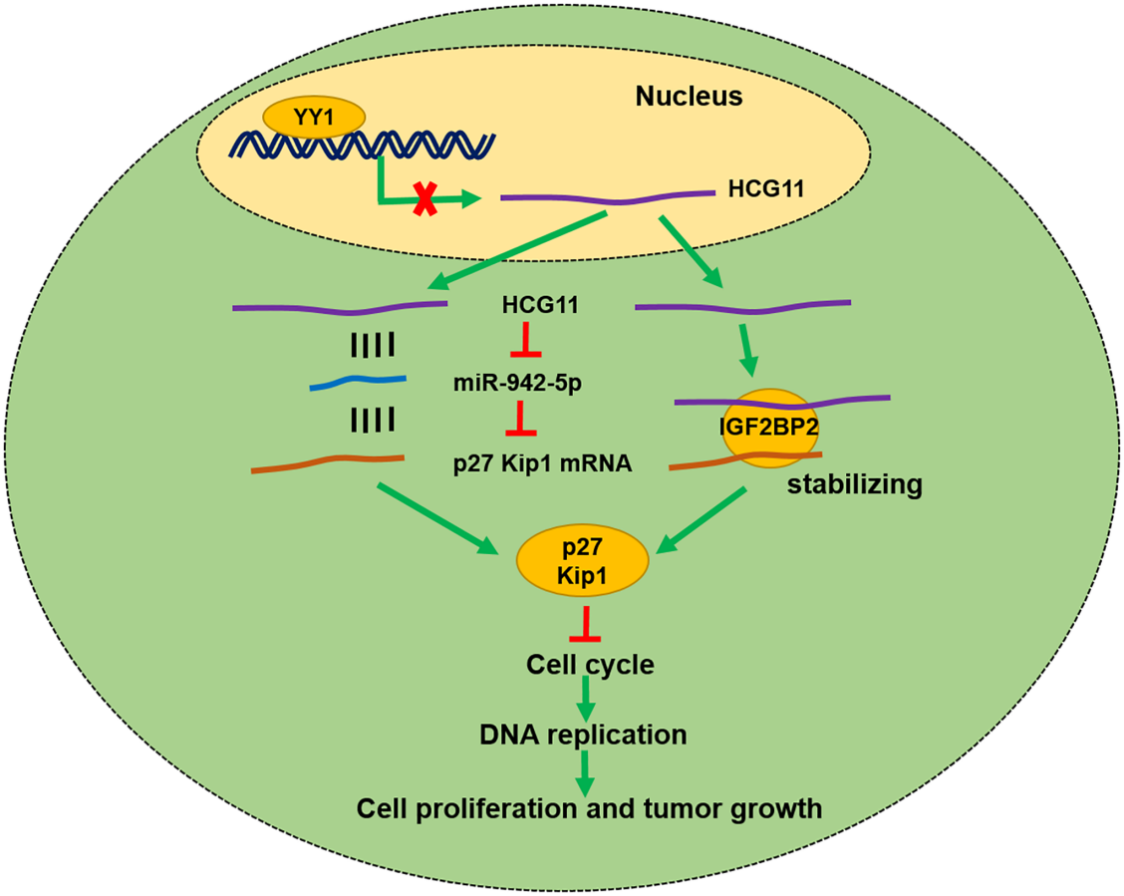
Schematic diagram showing HCG11 regulatory network in OS malignant process.

In conclusion, the present study proves that HCG11 is downregulated in OS and its low expression is correlated with larger tumor size and poor prognosis of OS. HCG11 restrains OS growth both *in vitro* and *in vivo* by raising the p27 Kip1 expression via binding to miR-942-5p and IGF2BP2 (Figure 7).

## MATERIALS AND METHODS

### Tissue samples

Samples of OS tissues and their matched adjacent non-tumor tissues (ANTs) were collected from 50 patients who underwent surgical resection at Beilun People’s Hospital. This study was approved by the Ethics Committee of the Beilun People’s Hospital and performed in line with the Declaration of Helsinki. Written informed consents were obtained from all participants.

### Cell culture and transfection

Five human OS cell lines (MG63, U2OS, HOS, 143B, and Saos-2) and one human osteoblast cell line (hFOB1.19) were purchased from American Type Culture Collection cell bank (ATCC, Manassas, VA, USA). Cells were cultured in RPMI-1640 media (HyClone, South Logan, UT, USA) supplemented with 10% fetal bovine serum (HyClone) in a humidified incubator containing 5% CO_2_ at 37°C. Cell transfection was conducted using Lipofectamine 3000 (Invitrogen, Carlsbad, MA, USA) according to the manufacturer’s instructions. Short hairpin RNAs (shRNAs) and overexpression plasmids for HCG11 and p27 Kip1, miR-942-5p mimics and inhibitors were obtained from GenePharma (Shanghai, China). Target sequences of shRNAs were presented in Supplementary Table 1.

### RNA extraction and qRT-PCR

Total RNAs were extracted from tissue samples and cell lines using TRIzol reagent (Invitrogen). RNAs (1 μg) were reversely transcribed into cDNAs using PrimeScript RT Reagent Kit (TaKaRa, Dalian, China). SYBR Premix Ex Taq (Takara) was applied to perform Real-time PCR analyses. β-actin and U6 served as internal controls. The relative gene expression was analyzed with the 2^−ΔΔCt^ method. Primers sequences: HCG11, forward, 5′-GCTCTATGCCATCCTGCTT-3′ and reverse, 5′-TCCCATCTCCATCAACCC-3′; miR-492-5p, forward, 5′-CTTCTCTGTTTTGGCCATGTG-3′ and reverse, 5′-CTCTACAGCTATATTGCCAGCCAC-3′; GAPDH, forward, 5′-GTTCCAATATGATTCCACCC-3′ and reverse, 5′-AGGGATGATGTTCTGGAGAG-3′; U6, forward, 5′-TCGCTTCGGCAGCACATA-3′ and reverse, 5′-TTTGCGTGTCATCCTTGC-3′.

### Protein extraction and western blotting

RIPA lysis buffer containing protease inhibitors was used to isolate total proteins. Equal amounts of total proteins were separated by SDS-PAGE gels and transferred onto PVDF membranes. The membranes were then incubated with primary antibodies against p27 Kip1 (1:1000, #3686, Cell Signaling Technology, Beverly, MA, USA) or β-actin (1:1000, #4970, Cell Signaling Technology) and secondary antibodies. Protein bands were detected by enhanced chemiluminescence.

### Cell counting kit-8 (CCK8) assay

Cells plated in 96-well plates were transfected with plasmids and oligonucleotides. After culturing for 24, 48 and 72 hours, the medium was replaced by 100 μL fresh RPMI-1640 medium containing 10% CCK-8 reagent (Dojindo, Kumamoto, Japan). After incubation at 37°C, the optical density values at 450nm were detected by a microplate reader.

### 5-Ethynyl-2’-deoxyuridine (EdU) assay

Cells plated in 6-well plates were transfected with plasmids and oligonucleotides for 48 hours. A Cell-Light EdU Apollo567 *In Vitro* Kit (Ribobio, China) was used to label cells with active DNA replication. Images were captured with a fluorescence microscope to calculate the proportion of the EdU-positive cells.

### Cell cycle analysis

Cells plated in 6-well plates were transfected with plasmids and oligonucleotides for 48 hours. A Cell Cycle and Apoptosis Analysis Kit (Beyotime, Shanghai, China) and Flow cytometry were used to analyze the cell cycle.

### Xenograft tumorigenesis model

BALB/c nude mice (male, 5 weeks old) were injected subcutaneously with 5 × 10^6^ OS cells stably HCG11 overexpressed or knocked-down. Tumor volumes were monitored every week (volume = width^2^ × length × 0.5). This study was approved by the Ethics Committee of the Beilun People’s Hospital and followed the Guide for the Care and Use of Laboratory Animals of the National Institutes of Health.

### RNA *in situ* hybridization

Alexa Fluor 488-labeled HCG11 probes and Cy3-labeled miR-942-5p probes were synthesized by Ribo-Bio (Guangzhou, China). The RNA *in situ* hybridization assay was performed using a Fluorescent *In Situ* Hybridization Kit (RiboBio). The nuclei were stained by DAPI. Images were captured with a fluorescence microscope.

### RNA pull-down assay

Probe-coated beads were prepared by incubating biotinylated HCG11 probe or NC probe (Ribo-Bio) with C-1 magnetic beads (Life Technologies). Then, cells were harvested and lysed, and the lysates were incubated with beads to pull-down RNA-RNA complexes. The complexes were then used to extract RNAs for further qRT-PCR and western blot analysis.

### RNA immunoprecipitation (RIP) assay

RIP assay was carried out with a Magna RIP RNA-Binding Protein Immunoprecipitation Kit (Millipore, Billerica, MA, USA). Briefly, the RIP buffer containing magnetic beads conjugated with IGF2BP2 antibody (11601-1-AP, Proteintech, Wuhan, China) or normal rabbit IgG was applied to immunoprecipitate the RNA-protein complexes. The complexes were then used to extract RNAs for further qRT-PCR analysis.

### Immunofluorescence

Cells were fixed, permeabilized and blocked before incubating with IGF2BP2 antibody (11601-1-AP, Proteintech). A CL488-conjugated Affinipure Goat Anti-Rabbit IgG was selected as the secondary antibody. Images were captured with a fluorescence microscope.

### Chromatin immunoprecipitation (ChIP) assay

The ChIP assay was performed using a Simple Chip Enzymatic Chromatin IP kit (Cell Signaling Technology) and YY1 antibody (#46395, Cell Signaling Technology) or normal rabbit IgG to immunoprecipitated the DNA-protein cross-links. Chromatin DNA collected were detected using qPCR assay with primers: forward, 5′-ACCGAAAAGTGCGCAAATCC-3′ and reverse, 5′-CGTTGTGCTGCAAATCTCCC-3′.

### Dual-luciferase reporter assay

The promoter sequences of HCG11 containing wild-type (wt) or mutant (mut) YY1 recognition sites were inserted into a PGL3-Basic luciferase reporter (Promega, Madison, WI, USA). The reporters were co-transfected into cells with YY1 overexpression plasmids. Luciferase activity was detected by a Dual Luciferase Reporter Gene Assay Kit (Beyotime, Shanghai, China).

The sequences of HCG11 or p27 Kip1 3′UTR containing wild-type or mutant miR-942-5p binding site were inserted into a pMIR-REPORT vector (Thermo Fisher Scientific, Waltham, MA, USA). The luciferase reporters were co-transfected into cells with miR-942-5p mimics. The luciferase activity was detected by a Dual Luciferase Reporter Gene Assay Kit (Beyotime).

### RNA stability analysis

Cells were incubated with 10 μg/mL actinomycin D (Sigma-Aldrich, St. Louis, USA) to block transcription, and then the p27 Kip1 mRNA levels at different time points were analyzed by qRT-PCR. The expression of 18S rRNA was selected as the internal control.

### Statistical analysis

All assays were performed for at least three times and data were presented as mean ± standard deviation (SD). Group comparisons were carried out with GraphPad Prism 5.0 (GraphPad Software, La Jolla, CA, USA) or SPSS 19.0 (IBM, Chicago, IL, USA) using Student’s *t* test, one-way analysis of variance or χ2 test. Survival analysis was performed using the Kaplan-Meier method and log rank test. Correlation was evaluated by Spearman’s analysis. *P* value < 0.05 was deemed statistically significant.

## Supplementary Materials

Supplementary Figure 1

Supplementary Table 1
