# Supplementary material for: lncRNA HCG11 suppresses human osteosarcoma growth through upregulating p27 Kip1
Source: Aging (Albany NY). 2021 Sep 13;13(17):21743–57. doi: 10.18632/aging.203517 (PMC8457558; doi:10.18632/aging.203517)
Supplement: Supplementary Figure 1 [file aging-13-203517-s001.pdf]

SUPPLEMENTARY FIGURE

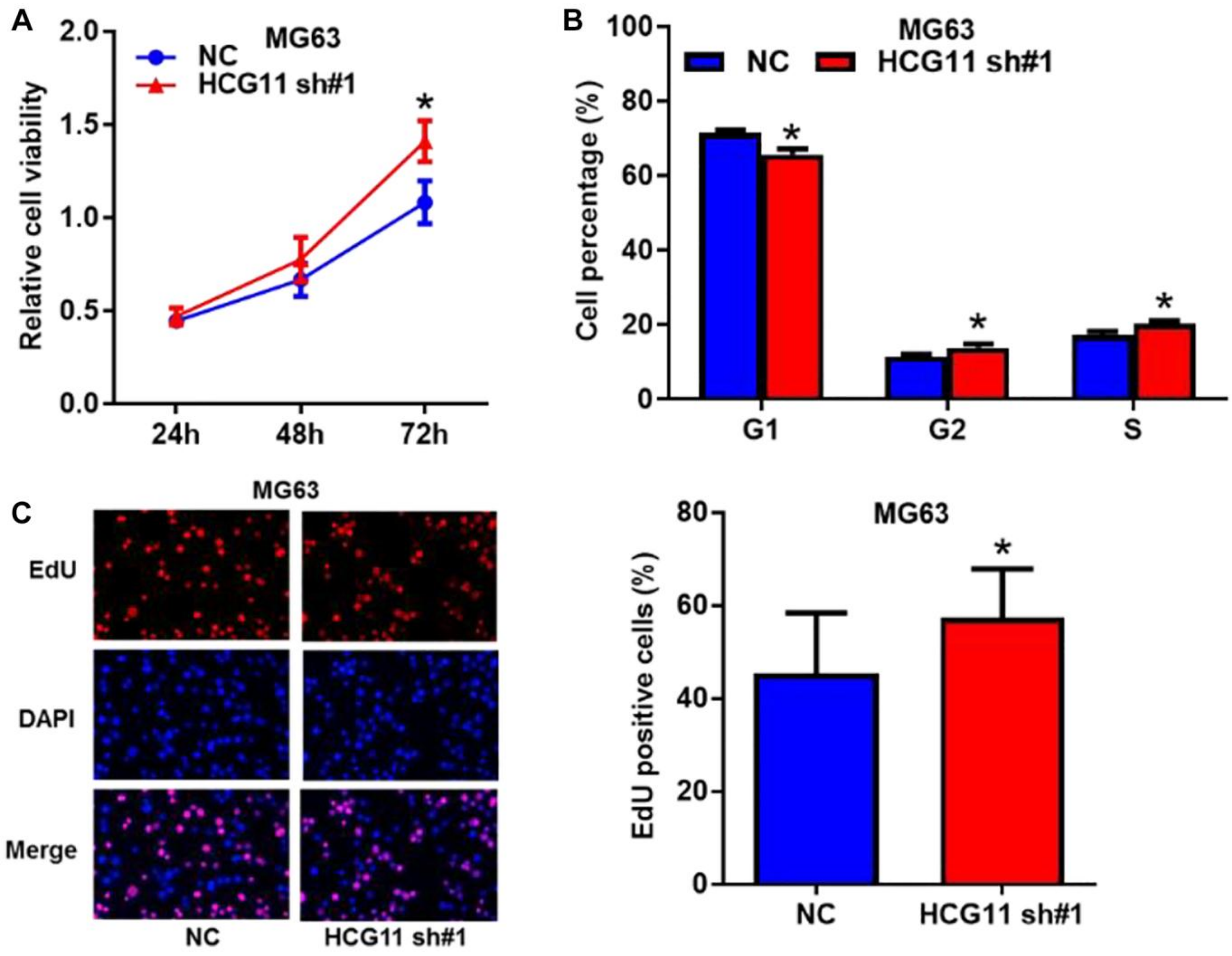

Supplementary Figure 1. Effects of HCG11 sh#1 on proliferation of MG63 cells. (**A**) Effect of HCG11 shRNA#1 on MG63 cells proliferation was assessed by CCK8 assays. (**B**) Effect of HCG11 shRNA#1 on the cell cycle of MG63 cells was analyzed by flow cytometry. (**C**) Effect of HCG11 shRNA#1 on the DNA replication of MG63 cells was assessed by EdU assay. $^{*}P < 0.05$.
