# Supplementary material for: lncRNA HCG11 suppresses human osteosarcoma growth through upregulating p27 Kip1
Source: Aging (Albany NY). 2021 Sep 13;13(17):21743–57. doi: 10.18632/aging.203517 (PMC8457558; doi:10.18632/aging.203517)
Supplement: Supplementary Table 1 [file aging-13-203517-s002.pdf]

SUPPLEMENTARY TABLE

Supplementary Table 1. Target sequences of shRNAs used in this study.

shRNAs	Target sequences (5′–3′)
HCG11 shRNA#1	GCAGAUUAGUCGAAUUCAA
HCG11 shRNA#2	CACAGAAGUUCAUGUUUAA
HCG11 shRNA#3	GAAGGAGCACAAAGUUCAA
YY1 shRNA	GAACUCACCUCCUGAUUAU
p27 Kip1 shRNA	UAGGAUAAGUGAAAUGGAUA
IGF2BP2 shRNA	GAUCUUUGGGAAACUGAAA
